# Association of Mitochondrial DNA Copy Number Variations with Triple-Negative Breast Cancer: A Potential Biomarker Study

**DOI:** 10.3390/diseases13060175

**Published:** 2025-06-01

**Authors:** Karin Manto, Sevdican Ustun Yilmaz, Zeliha Pala Kara, Halil Kara, Fatma Tokat, Cemaliye B. Akyerli, Cihan Uras, Meltem Muftuoglu, Ugur Özbek

**Affiliations:** 1 Department of Genome Studies, Institute of Health Sciences, Acibadem Mehmet Ali Aydinlar University, Atasehir, 34638 Istanbul, Turkey; 2 Department of Medical Biotechnology, Institute of Health Sciences, Acibadem Mehmet Ali Aydinlar University, Atasehir, 34638 Istanbul, Turkey; 3 Department of Pharmacology, Faculty of Pharmacy, Istanbul University, Beyazit, 34452 Istanbul, Turkey; 4 Department of General Surgery, School of Medicine, Acibadem Mehmet Ali Aydinlar University, Atasehir, 34638 Istanbul, Turkey; 5 Department of Pathology, School of Medicine, Acibadem Mehmet Ali Aydinlar University, Atasehir, 34638 Istanbul, Turkey; 6 Department of Medical Biolgoy, School of Medicine, Acibadem Mehmet Ali Aydinlar University, Atasehir, 34638 Istanbul, Turkey; 7 Department of Molecular Biology and Genetics, Faculty of Engineering and Natural Sciences, Acibadem Mehmet Ali Aydinlar University, Atasehir, 34638 Istanbul, Turkey; 8 Izmir Biomedicine and Genome Center (IBG), 35340 Izmir, Turkey

**Keywords:** mtDNA, TNBC, biomarkers, copy number variation, qPCR, neoadjuvant chemotherapy

## Abstract

Background/Objectives: Triple-negative breast cancer (TNBC) is a highly aggressive subtype with limited therapeutic options, and identifying reliable biomarkers for diagnosis and prognosis is crucial for improving patient outcomes. Mitochondrial DNA (mtDNA) copy number has been linked to an increased risk of developing various types of cancer, including breast cancer. However, there is a lack of understanding regarding how mtDNA copy number variations may influence the development and progression of TNBC. Methods: This study investigated mtDNA copy number in TNBC tumors and corresponding normal breast tissues from 23 TNBC patients who received neoadjuvant chemotherapy. The relative mtDNA copy number was estimated using quantitative PCR for the NADH dehydrogenase subunit 1 (ND1) and subunit 5 (ND5) regions. Results: The results showed a significant decrease in mtDNA copy number in TNBC tumor tissues compared to corresponding normal breast tissue. However, no significant correlation was found between mtDNA content and clinical parameters such as age, tumor size, or chemotherapy response. Conclusions: These results suggest that while mtDNA content decreases in TNBC tumors, it may not directly influence these clinical characteristics. Despite some inconsistencies in the literature regarding mtDNA dynamics in cancer, this study supports the potential of mtDNA as a biomarker for TNBC. Larger cohort studies are needed to further validate these results and explore the role of mtDNA in guiding personalized treatment strategies for TNBC patients.

## 1. Introduction

Triple-negative breast cancer (TNBC) is an aggressive subtype of breast cancer, accounting for approximately 15% of global breast cancer cases [1,2]. TNBC is defined by the absence of estrone receptor (ER), progesterone receptor (PR), and HER2 expression, which are typically targeted by breast cancer treatments [1,2,3]. The absence of these receptors severely restricts the therapeutic choices available for TNBC since it prevents the use of hormone therapy or HER2-targeted treatments [2]. Additionally, TNBC is characterized by early relapse and a high propensity to metastasize to vital organs such as the liver, lungs, and central nervous system [3]. Therefore, compared to individuals with other subtypes of breast cancer, TNBC patients frequently have a worse prognosis. Since traditional targeted therapies are ineffective for TNBC patients, chemotherapy is the main treatment option.

For early-stage TNBC, neoadjuvant chemotherapy is the gold standard, allowing for an assessment of tumor response and personalized treatment regimens [4]. This approach, often combined with surgery, is effective in reducing tumor size and lymph node involvement, thereby improving the chance of breast-conserving surgery, particularly for tumors larger than 2 cm. Neoadjuvant chemotherapy also reduces the chance of long-term recurrence, promotes faster recovery, and lessens the invasiveness of surgery [5].

Mitochondrial DNA (mtDNA) alterations, such as point mutations, deletions, and rearrangements, are known to have a significant role in the development and production of cancer [6]. Although these variations have been observed across various cancers, their precise role in disease progression remains unclear [6]. Some studies propose that mtDNA mutations result from the clonal expansion of pre-existing heteroplasmic polymorphisms during cancer progression, whereas others suggest they contribute to cancer development by generating truncated proteins that disrupt normal cellular functions [7].

mtDNA copy number variations (CNVs) exhibit fluctuations in cancer as a result of diverse cellular and environmental factors [8]. Alterations within the mtDNA D-loop region can result in a decrease in mtDNA copy numbers, whereas increased mtDNA levels have been linked to higher levels of oxidative stress in cancer cells [8]. Numerous studies have investigated the relationship between blood mtDNA levels and cancer risk. Increased mtDNA levels have been associated with a higher risk of developing cancers such as papillary thyroid, colorectal, ovarian, lung, prostate, head, and neck cancers. In contrast, decreased mtDNA levels have been reported in cancers such as bone, kidney, liver, and breast cancer. However, higher and lower levels of mtDNA in peripheral blood were associated with an increased risk of developing colorectal carcinoma and breast cancer [7,8,9,10,11,12].

These conflicting findings probably result from variations in methodologies, sample size, and investigated cancer types, as well as limited knowledge regarding tissue-specific mtDNA changes within the same individual [12]. Despite these inconsistencies, the association between mtDNA alterations and cancer severity has generated interest in mtDNA CNVs as potential biomarkers for cancer detection and prognosis [13]. Increased mtDNA levels may protect tumor cells from apoptosis, while low mtDNA levels can promote reactive oxygen species (ROS) generation, potentially enhancing tumor susceptibility to chemotherapy [13,14]. This suggests that mtDNA CNVs could influence survival outcomes and treatment responses in various cancers.

Previous research investigating mtDNA content in breast cancer has examined its association with tumor phenotype, drug response, and prognosis [10,15]. Most findings imply that breast tumor tissues exhibit lower mtDNA content compared to healthy tissues [11,15,16]. However, Lin et al. have reported increased mtDNA content in breast cancer tissues [17]. These inconsistencies indicate that further research is required to clarify mtDNA’s potential as a reliable biomarker in breast cancer.

TNBC is known to exhibit unique metabolic reprogramming and mitochondrial alterations compared to other breast cancer subtypes [18]. Given the central role of mitochondria in energy metabolism and apoptosis, analyzing mtDNA copy number in TNBC could provide novel insights into tumor biology and progression [18]. Specifically, TNBC tumors demonstrate lower mtDNA copy numbers, impaired oxidative phosphorylation, and altered expression of mitochondrial transporters and biogenesis regulators, such as SLC25A25 [9]. These alterations contribute to the Warburg effect and support aggressive tumor growth, chemoresistance, and metastatic spread [18]. Furthermore, tumors with low mtDNA content harbor a higher frequency of pathogenic BRCA1 mutations, suggesting a link between nuclear genome instability and mitochondrial genome maintenance [9,18].

Additionally, chronic inflammation, which has been implicated in breast cancer pathogenesis, may contribute to changes in mtDNA copy number through oxidative stress and mitochondrial dysfunction [18].

While mtDNA copy number variations have been studied in breast cancer, there is a lack of research specifically focusing on TNBC. Investigating mtDNA as a biomarker in TNBC could provide new insights into tumor biology and risk stratification, as well as potentially improve treatment strategies for TNBC patients. Thus, the purpose of this study is to explore the relationship between mtDNA CNVs and TNBC. Using real-time quantitative PCR (qPCR) to analyze mtDNA and nuclear DNA (nDNA) from formalin-fixed paraffin-embedded (FFPE) tissues, we aim to determine whether mtDNA CNVs can serve as biomarkers in TNBC patients undergoing neoadjuvant chemotherapy. These findings may provide critical insights into the clinical utility of mtDNA CNVs in TNBC management.

## 2. Materials and Methods

### 2.1. Study Participants

Twenty-three TNBC tissues from patients who had received neoadjuvant chemotherapy and non-cancerous normal breast tissue from the same individual were obtained from the Pathology and General Surgery Departments at Acibadem Maslak Hospital, Istanbul, Turkey. FFPE tru-cut biopsy samples were collected retrospectively. The evaluation and grading of the TNBC and corresponding normal tissues were performed by the Pathology Department of Acibadem Maslak Hospital, according to the published criteria [19]. All patients received standard neoadjuvant chemotherapy regimens appropriate for TNBC, which commonly included anthracycline-based and taxane-based agents. Written informed consents were obtained from all patients before participating in this study. Ethical approval for this study was obtained from the Acibadem University and Acibadem Healthcare Institutions Medical Research Ethics Committee (Decision No: 2022-16/01). All procedures adhered to institutional guidelines, and patient privacy was provided through anonymization and coding of samples.

### 2.2. DNA Isolation from FFPE Tissue

Total DNA was extracted from FFPE tissue sections using the QIAamp DNA FFPE Tissue Kit (QIAGEN, Hilden, Germany), according to the manufacturer’s protocol, with minor modifications implemented to maximize yield. Specifically, the proteinase K treatment duration was extended from 1 h to 3 h [20], and the elution buffer was replaced with ddH_2_O, which was applied five times until the total volume reached 100 µL instead of a single application. DNA concentration and purity were evaluated using a NanoDrop Scientific™ 1000 spectrophotometer (Thermo Fisher, Wilmington, DE, USA). Concentration was determined by measuring absorbance at 260 nm, while purity was assessed using the 260/280 nm ratio, with values ≥ 1.60 considered acceptable for FFPE-derived DNA [21,22,23]. The concentration and purity values for each sample are provided in Supplementary Table S1 (Table S1), demonstrating adequate DNA quality for downstream qPCR analysis.

### 2.3. Quantification of mtDNA and nDNA

Variations in mtDNA copy number were evaluated using qPCR on a Bio-Rad CFX Real-Time PCR system. This approach enables the relative quantification of mtDNA copy number changes. Three primer sets were used: 18S rRNA primers (forward primer (F): 5′ TAGAGGGACAAGTGGCGTTC 3′; reverse primer (R): 5′ CGCTGAGCCAGTCAGTGT 3′) for nuclear DNA (nDNA) and ND1 (F: 5′ CCCTAAAACCCGCCACATCT 3′; R: 5′ GAGCGATGGTGAGAGCTAAGGT 3′) and ND5 (F: 5′ CCGGAAGCCTATTCGCAGGA 3′; R: 5′ ACAGCGAGGGCTGTGAGTTT 3′) primers for mtDNA.

The qPCR reactions were carried out using a Bio-Rad CFX Real-Time PCR system (BioRad, Hercules, CA, USA). Each reaction contained 50 ng of DNA, 10 µL of 2X SYBR Green qPCR Mix (QPSY02, GeneMark, Taichung, Taiwan), and 0.2 µM of forward and reverse primers in a final volume of 20 µL. The thermal cycling protocol consisted of an initial denaturation at 95 °C for 3 min, followed by 40 cycles of denaturation at 95 °C for 10 s, annealing at 59 °C for 10 s, and extension at 72 °C for 20 s. A melting curve analysis was performed at the end, with a temperature range of 65 °C to 95 °C, incrementing by 0.5 °C every 5 s.

The mtDNA content was determined by calculating the threshold cycle (Ct) values obtained from qPCR for both healthy and tumor tissue samples. The relative mtDNA copy number was quantified using the formula 2 × 2^ΔCt^, where ΔCt = CtnDNA − CtmtDNA, representing the difference between the Ct values of nDNA and mitochondrial DNA [24]. Each sample was analyzed in duplicate to ensure accuracy, and the average Ct values from these replicates were used for the final calculations.

### 2.4. Statistical Analysis

Statistical analyses were conducted using GraphPad Prism 10.2.2 and R (version 4.3.2). Differences in mtDNA content between TNBC FFPE tumor samples and their corresponding healthy tissues were evaluated using the Wilcoxon matched-pairs signed-rank test. For correlation analysis, both Pearson’s and Spearman’s correlation tests were performed. The Pearson correlation coefficient was used to assess the strength of linear relationships between normally distributed variables, while the Spearman correlation test was employed as a nonparametric measure of association, ranking data without requiring assumptions about their distribution. The Kruskal–Wallis test was also used to assess mtDNA variations across different groups, including tumor stages (T-stage), N-stage, histological grading, and other clinical factors. A significance threshold of *p* < 0.05 was applied, with all statistical tests performed as two-sided analyses.

### 2.5. Use of Generative AI Tools

During the preparation of this manuscript, the authors used ChatGPT (OpenAI, GPT-4, version: GPT-4o-mini; accessed April 2025) to assist in language refinement, grammar correction, and clarity improvements during manuscript writing. All scientific content, study design, data interpretation, and collusions were generated and verified independently by the authors.

## 3. Results

### 3.1. Characteristics of TNBC Patients

The characteristics of the 23 TNBC patients are presented in Table 1. The mean age of the patients was 48.8 ± 10.4 years, with a range of 31 to 69 years. Among the patients, 21 of them were under 65 years old, while 2 were over 65 years. Tumor stages were classified as T1 (n = 4), T2 (n = 15), T3 (n = 3), and T4 (n = 1). Lymph node involvement was distributed as N0 (n = 8), N1 (n = 8), N2 (n = 2), and N3 (n = 3). Distant metastasis was observed in only one patient (M1), while 22 patients showed no evidence of distant metastasis (M0). Treatment response was categorized as complete (n = 13) and partial (n = 10). The Ki67 proliferation index was also assessed for each tumor tissue. Among the 23 TNBC patients, none exhibited low Ki67 expression (<15%), while all 23 showed high Ki67 expression (≥15%) 20]. Histologically, the majority of tumors were classified as invasive ductal carcinoma (IDC) (n = 22), with one case identified as metaplastic carcinoma. The primary tumor sizes ranged from 14 mm to 100 mm, with a median size of 26 mm. Tumor stages were as follows: Stage 1 (n = 1), Stage 2 (n = 14), and Stage 3 (n = 8). Histological grading revealed that 20 patients had grade 3 tumors, while 2 patients had grade 2 tumors. For nuclear grading, 21 patients had grade 3 tumors, while 2 patients had grade 2 tumors.

### 3.2. Experimental and Statistical Analysis

qPCR analysis was conducted to determine the mtDNA content in both TNBC tissues and their corresponding normal tissues from 23 TNBC samples. For ND1, the mtDNA content in tumor tissues was lower than that in corresponding normal tissues in 18 of the 23 samples. The average mtDNA content ranged from 8.65 to 159.71 in normal tissues and 3.10 to 53.14 in tumor tissues (Supplementary Table S2). Similarly, for ND5, tumor tissues exhibited decreased mtDNA content compared to normal tissues in 18 of 23 samples, with average mtDNA content ranging from 2.70 to 50.35 in normal tissues and 0.82 to 25.16 in tumor tissues (Supplementary Table S3).

**Figure 1:**
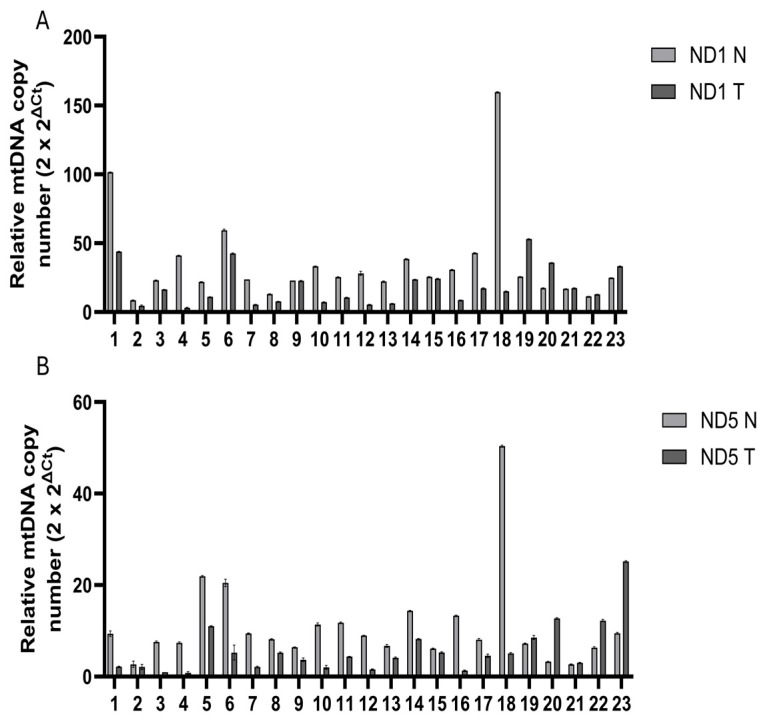
Relative mtDNA copy number content in TNBC samples for ND1 and ND5: (**A**) ND1-based mtDNA copy number variations; (**B**): ND5-based mtDNA copy number variations. The bar graph represents the content of mtDNA in both corresponding normal (N) and tumor TNBC (T) tissues for each sample [25].

The relative mtDNA copy number content for both ND1 and ND5 is shown in Figure 1, highlighting a consistent decrease in tumor tissues compared to normal tissues across 18 samples.

**Figure 2:**
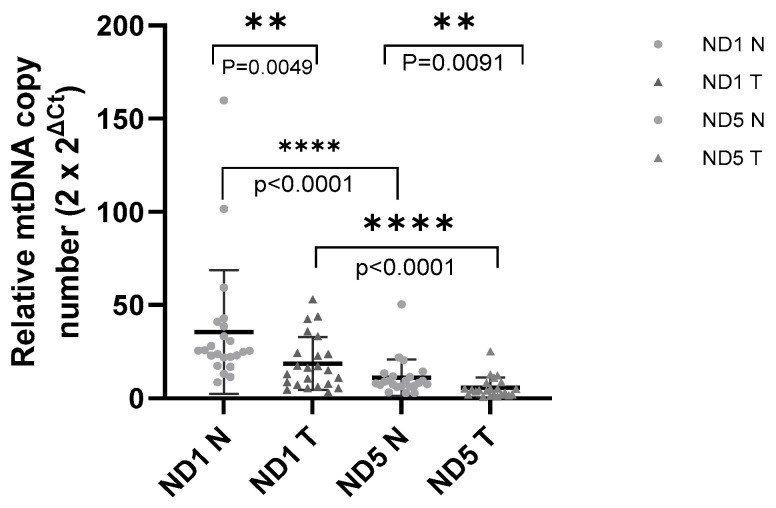
Relative mtDNA copy number variations in samples: relative mtDNA copy number was determined by the 2 × 2^ΔCt^ method and compared between values (mean ± SD) of the patient’s corresponding normal tissue and tumor tissue. Student’s *t*-test (unpaired, two-tailed) was performed to analyze the significance. ** (*p* < 0.01); **** (*p* < 0.0001) [25].

The Wilcoxon matched-pairs signed-rank test was used to compare mtDNA levels between normal and tumor tissues across 23 TNBC sample pairs. Of these, 18 pairs demonstrated significantly higher mtDNA levels in corresponding normal tissues compared to tumor tissues (*p* < 0.05) (Figure 2).

Correlation analysis of mtDNA content measured with ND1 and ND5 primers was performed for both normal and tumor tissues using Pearson’s and Spearman’s correlation coefficients (Table 2). The findings demonstrated a statistically significant correlation (*p* ˂ 0.05) between the ND1 and ND5 mtDNA content in both tissue types, highlighting the consistent association between the two primers in all samples. Additionally, analysis of mtDNA content changes across all samples revealed statistically significant results only in cases with decreasing mtDNA content. Samples with increasing mtDNA content showed no statistical significance (Table 2).

The association between mtDNA content and clinical parameters, including age, response to neoadjuvant chemotherapy, and Ki67 status was analyzed using Pearson’s and Spearman’s correlation coefficients. These results are summarized in Table 3, Table 4, and Table 5, respectively. No statistically significant correlation was observed between mtDNA content and age (Table 3) or response to chemotherapy (Table 4) across the analyzed samples. Similarly, no statistically significant correlations were found between mtDNA and Ki67 status for ND1 or ND5, regardless of whether all samples or only those with decreasing mtDNA content were considered (Table 5).

The Kruskal–Wallis test was employed to examine the relationship between mtDNA differences and various clinical parameters, including tumor T and N stages, overall stage, histological grading, nuclear grading, and treatment response (Table 6 and Table 7). The results revealed no statistically significant differences (*p* > 0.05) in mtDNA content across these variables. For example, mtDNA differences for both ND1 and ND5 were not statistically significantly associated with tumor stage, histological grading, or nuclear grading. Similarly, no statistically significant association was found between mtDNA differences and treatment response.

## 4. Discussion

TNBC accounts for approximately 15% of all breast cancer cases [1,2] and is characterized by the simultaneous absence of ER, PR, and HER-2 expression. This aggressive subtype is associated with poor prognosis and limited treatment options compared to other breast cancer subtypes [1,2,3]. Despite advancements in diagnostic and therapeutic strategies that have improved outcomes in developed countries, TNBC continues to pose significant challenges, particularly in developing nations [12]. Exploring novel biomarkers, such as mtDNA CNV, may provide opportunities for developing targeted therapies and improved patient care [26].

mtDNA plays a critical role in cellular metabolism, apoptosis, and oxidative stress balance. A decrease in mtDNA copy number is associated with mitochondrial dysfunction, which disrupts ATP production via oxidative phosphorylation and increases the generation of ROS due to the impaired activity of the electron transport chain (ETC) [27]. The increase in ROS can damage mitochondrial components, including mtDNA itself, which can lead to a continuous cycle of mitochondrial dysfunction and oxidative stress [27].

The relationship between mtDNA content and cancer risk has long been studied. However, the role of mtDNA content varies across cancer types, influenced by various factors including mitochondrial activity [12,28]. Decreased mtDNA content is associated with apoptotic resistance, which is a critical factor in cancer cell survival. This alteration can inhibit the mitochondrial-mediated cell death pathway, enabling malignant cells to escape apoptosis and keep proliferating [29]. Some studies have also shown that decreased mtDNA copy number is related to the promotion of epithelial–mesenchymal transition, especially in TNBC. This process may facilitate metastasis and poor clinical outcomes [30]. Numerous studies have explored mtDNA copy number changes in breast cancer, analyzing both paired tissue and blood samples, yet the findings often conflict, underscoring the complexity of the relationship between mtDNA copy number changes and breast cancer [12]. Furthermore, while previous studies have focused on the general association between mtDNA content and breast cancer, there remains a notable lack of literature concerning the specific relationship between mtDNA CNVs and TNBC in patients who have received neoadjuvant chemotherapy. To address this gap, the present study investigated mtDNA CNVs in paired tumor and normal tissues from TNBC patients who received neoadjuvant chemotherapy.

One of this study’s main limitations is the relatively small sample size, which may reduce the statistical power and generalizability of the findings. In the initial stage, we planned to conduct a cohort of 100 patients; however, due to its retrospective design, many archived samples lacked sufficient tissue or yielded inadequate DNA for reliable analysis. Consequently, the final cohort size was limited to 23 patients. Future studies involving larger patient cohorts are essential to validate the potential role of mtDNA copy number as a biomarker in TNBC.

Total DNA was extracted from FFPE tumor tissues and their corresponding normal tissues from these TNBC patients prior to undergoing neoadjuvant chemotherapy. The extracted DNA was subsequently used to quantify mtDNA CNVs through SYBR-green-based qPCR. FFPE tissue preservation aims to maintain cellular architecture and components; however, prolonged formalin fixation poses challenges, including protein–nucleic acid crosslinking and random nucleotide sequence breakages [21,31]. Moreover, there is no universally accepted standard for tissue fixation, and even minor alterations in fixation protocols can significantly impact the quality and yield of extracted DNA [21]. Although our results generally meet satisfactory standards for FFPE samples, inherent heterogeneity and possible contamination between tumor and normal tissues from tru-cut biopsy specimens are still important factors to take into account. Even though single-source samples provide higher accuracy, careful validation procedures are necessary because of the possibility of tumor tissue infiltration into normal tissue. In this context, DNA concentration and purity were assessed for all samples, and the values were within acceptable ranges for FFPE-derived nucleic acids, ensuring the reliability of qPCR-based mtDNA copy number quantification (Table S1).

Although our study revealed a consistent reduction in mtDNA content, prior studies revealed variable correlations with clinical features such as age, tumor grade, hormone receptor status, and metastasis, suggesting that further studies are needed to clarify these inconsistencies. A study examining mtDNA copy number in 59 breast cancer cases found significantly lower mtDNA content in tumor tissues, with a notable association with older age and higher histological grade [16]. Similarly, a study by Cheng Fan A.X. et al. involving 51 breast cancer patients reported decreased mtDNA content in 82% of tumor tissues. However, no correlation was found between mtDNA content and age, although a relationship with hormone receptor status was noted. Furthermore, no significant correlation was observed between clinical parameters such as tumor size, lymph node involvement, metastasis, and histological grade [32]. Mabo et al. also reported no correlation between mtDNA content and tumor grade or metastasis. Additionally, several studies have demonstrated lower mtDNA content in breast cancer tissues compared to corresponding normal tissues [33,34]. These findings align with our results showing decreased mtDNA content in TNBC tumor tissues, but they contrast with the associations observed with clinicopathological features. In our cohort, this may be explained by the limited sample size, the specificity of the TNBC subtype, or methodological factors such as DNA extraction from FFPE tissue specimens.

Similarly, while Lin et al. observed a positive correlation between mtDNA content and Ki67 expression in IDC, our findings showed no such association, likely due to our patient group uniformly exhibiting high Ki67 expression. They also reported that advanced T-status was linked to higher mtDNA copy ratios and increased rates of mtDNA D310 mutations in IDC [17]. However, consistent with our results, they did not observe any significant correlation between N-status, cancer stage, histological grade, and mtDNA copy number [17]. These discrepancies indicate that mtDNA dynamics may vary not only among different cancer subtypes, but also due to differences in methodology, patient characteristics, and interindividual variation. In our cohort, the uniformly high Ki67 index may have masked any potential correlation with mtDNA content, indicating that the biological behavior of TNBC may not always follow the same trends observed in IDC.

Few studies have indicated an association between a high mtDNA copy number and an increased risk of developing breast cancer. A pilot study of 103 breast cancer patients revealed that a high mtDNA copy number was associated with a significantly increased risk of breast cancer compared to a low copy number [35]. Furthermore, mtDNA copy number has a significantly negative association with several crucial endogenous oxidants and antioxidants present in the blood [36]. While increased mtDNA content has been associated with a higher breast cancer risk in some populations [35,37], our study highlights that, in the context of TNBC, particularly in tumor tissue, mtDNA copy number is more frequently decreased. This contrast underscores the heterogeneity of breast cancer and the importance of molecular and subtype-specific contexts.

Another study by Hsu et al. explored the relationship between mtDNA content and drug response, suggesting that lower mtDNA content is associated with increased drug sensitivity and higher ROS production during doxorubicin treatment [15]. Furthermore, reduced mtDNA content can result in decreased oxidative phosphorylation capacity under hypoxic conditions during cancer development and progression [32]. When oxidative stress exceeds a certain threshold, it can trigger an apoptotic program that kills tumor cells [15]. Therefore, biochemical characteristics associated with low mtDNA content may serve as potential biomarkers for predicting patient outcomes following ROS-generating chemotherapy [15]. This aligns with our finding of reduced mtDNA copy number in TNBC tumor tissues, which may point to enhanced oxidative stress and possible chemosensitivity in specific subgroups, although there is no observation of a direct correlation with treatment response. Moreover, in cancer cells, such as breast cancer cells, mitochondrial dysfunction is a key feature that supports the metabolic reprogramming known as the Warburg effect, a process in which cells shift toward glycolysis even in the presence of oxygen [27,29]. Although no correlation between mtDNA content and treatment response was observed in this study, the reduction in mtDNA copy number in TNBC tumor tissues may reflect this metabolic shift. Altered mtDNA levels could therefore represent a molecular adaptation to the unique metabolic requirements of aggressive tumors such as TNBC, supporting their growth and survival under stress conditions.

In other cancer types, mtDNA copy number exhibits varying characteristics. In colorectal cancer, cervical cancer, osteosarcoma, and lung cancer, the mtDNA copy number was found to be lower in tumor tissues [16,28,38,39,40,41]. In contrast, in endometrial adenocarcinoma and acute lymphoblastic leukemia, the mtDNA copy number was found to be increased [28,41,42].

This study focused on the ND1 and ND5 genes of mtDNA; however, some studies have used different primers and methods to amplify various regions of mtDNA, such as the D-loop, cytochrome c oxidase subunit I [36], and *MTATP 8* gene [32]. Yu M. et al. demonstrated that tumors with mutations in the D-loop exhibit lower mtDNA content compared to those without such mutations [16]. Somatic mutations in the D-loop region are significant contributors to decreased mtDNA levels in breast tumors [16]. Further studies examining different mtDNA regions, particularly the D-loop, could provide additional insights.

It is important to note that several studies have reported conflicting results; however, our results align with the existing body of literature. While this study consistently shows a decrease in mtDNA copy number in cancerous tissue compared to normal tissue, other studies have observed higher mtDNA content in cancerous tissues, underscoring the complexity of mtDNA dynamics in cancer [35,37,38]. Furthermore, the variability in clinical parameters highlights the need for further research. This study showed no significant association between age and mtDNA content, suggesting that age may not be a key factor in mtDNA alterations, as observed in the study by Cheng Fan A.X. et al. [32]. Similarly, our results showed no correlation between mtDNA content and response to neoadjuvant chemotherapy, indicating that mtDNA dynamics may not directly influence treatment outcomes in TNBC patients. However, the absence of post-neoadjuvant chemotherapy samples, due to limited material resources, restricts our analysis. Comparing mtDNA content before and after treatment could offer valuable insights into treatment response.

## 5. Conclusions

In conclusion, the management of TNBC remains a significant challenge due to its aggressive nature and limited treatment options. Our study, focusing on mtDNA CNV in TNBC patients undergoing neoadjuvant chemotherapy, provides valuable insights into the potential of mtDNA as a biomarker for this aggressive subtype. While our results align with previous research showing decreased mtDNA content in tumor tissues compared to normal tissues, the lack of significant correlation between mtDNA copy number, age, chemotherapy response, and other clinical parameters suggests that mtDNA dynamics may not directly influence these clinical parameters. The presence of conflicting evidence in the literature further emphasizes the complexity of mtDNA’s role in cancer. Based on our preliminary findings, our study can be considered as a pilot study that may guide future research focused on understanding the mechanisms and clinical relevance of mtDNA alterations in TNBC. Given that the analysis involved neoadjuvant chemotherapy-treated TNBC patients and compared tumor tissues with matched normal tissues, the study’s significance is further underscored by its potential to reveal treatment-related mtDNA dynamics. Despite the modest sample size, our study highlights the potential of mtDNA as a biomarker for TNBC, and larger studies are needed to validate these findings and explore its clinical utility in treatment strategies for TNBC patients.

## Figures and Tables

**Table 1:** Clinical and pathological characteristics of TNBC patients [25].

Characteristics	Patients (n = 23)
Age range (years)	32–69
Mean (years)	49.22 ± 10.24
<65 years	21
>65 years	2
T-stage	T1 (4); T2 (15); T3 (3); T4 (1)
N-stage	N0 (8); N1 (8); N2 (2); N3(5)
M-stage	M0 (22); M1 (1)
Ki67 status (%)	Low (<15%): 0; High (≥15%): 23
Histological type	IDC (22); Metaplastic carcinoma (1)
Primary tumor size (mm)	14–100 (Median: 26)
Stage	1 (1); 2 (14); 3 (8)
Histological grade	2 (2); 3 (20); NA (1)
Nuclear grade	2 (2); 3 (21)
Treatment response	Complete (13); Partial (10)
Control (n) *	23

NA: not applicable. * Since control tissues are from the same individual (the corresponding normal tissue), their number and age properties are identical to those of TNBC tissue.

**Table 2:** Correlation of mtDNA content in corresponding normal and tumor tissue between ND1 and ND5 [25].

		Pearson		Spearman	
		r *	*p*-Value **	r *	*p*-Value **
All samples (n = 23)	ND1N vs. ND5N	0.81	<0.0001	0.59	0.003
	ND1T vs. ND5T	0.40	0.06	0.60	0.003
Decreasing mtDNA content (n = 18)	ND1N vs. ND5N	0.91	<0.0001	0.43	0.072
	ND1T vs. ND5T	0.67	0.002	0.62	0.006
Increasing mtDNA content (n = 5)	ND1N vs. ND5N	0.55	0.33	0.60	0.35
	ND1T vs. ND5T	0.15	0.81	0.10	0.95

* r is the correlation coefficient. ** *p* is a statistical significance of association, (*p* < 0.05).

**Table 3:** Correlation of mtDNA content with response to age (n = 23) [25].

	Pearson		Spearman	
	r *	*p*-Value **	r *	*p*-Value **
Age vs. ND1N	0.12	0.59	−0.05	0.83
Age vs. ND1T	−0.08	0.71	−0.20	0.35
Age vs. ND5N	0.05	0.83	0.08	0.70
Age vs. ND5T	−0.05	0.83	0.01	0.94

* r is the correlation coefficient. ** *p* is a statistical significance of association, (*p* < 0.05).

**Table 4:** Correlation of mtDNA content with response to neoadjuvant chemotherapy response (n = 23) [25].

	Pearson		Spearman	
	r *	*p*-Value **	r *	*p*-Value **
Response vs. ND1N	0.09	0.67	0.06	0.76
Response vs. ND1T	−0.09	0.68	0.01	0.95
Response vs. ND5N	0.29	0.18	0.17	0.43
Response vs. ND5T	0.25	0.25	0.26	0.22

* r is the correlation coefficient. ** *p* is a statistical significance of association, (*p* < 0.05).

**Table 5:** Correlation of mtDNA content with response to Ki67 status.

	Pearson		Spearman	
	r *	*p*-Value **	r *	*p*-Value **
Ki67 vs. ND1 mtDNA difference (n = 23)	0.15	0.48	0.04	0.85
Ki67 vs. ND5 mtDNA difference (n = 23)	0.10	0.64	0.10	0.62
Ki67 vs. ND1 mtDNA difference (decreasing) (n = 18)	0.20	0.43	0.17	0.48
Ki67 vs. ND5 mtDNA difference (decreasing) (n = 18)	0.16	0.52	0.29	0.24

* r is the correlation coefficient. ** *p* is a statistical significance of association, (*p* < 0.05).

**Table 6:** Results of the Kruskal–Wallis test for mtDNA differences.

Variable	Grouping Variable	Chi-Squared Statistic	Degrees of Freedom (df)	*p*-Value *
mtDNA difference (ND1)	Tumor T stage	3.79	3	0.28
mtDNA difference (ND5)	Tumor T stage	5.01	3	0.17
mtDNA difference (ND1) (decreasing)	Tumor T stage	4.03	3	0.26
mtDNA difference (ND5) (decreasing)	Tumor T stage	1.70	3	0.63
mtDNA difference (ND1)	Tumor N stage	0.71	3	0.87
mtDNA difference (ND5)	Tumor N stage	0.85	3	0.84
mtDNA difference (ND1) (decreasing)	Tumor N stage	1.65	3	0.65
mtDNA difference (ND5) (decreasing)	Tumor N stage	1.02	3	0.80
mtDNA difference (ND1)	Stage	2.08	2	0.35
mtDNA difference (ND5)	Stage	2.13	2	0.34
mtDNA difference (ND1) (decreasing)	Stage	3.45	2	0.18
mtDNA difference (ND5) (decreasing)	Stage	2.53	2	0.28
mtDNA difference (ND1)	Histological grading	1.24	2	0.54
mtDNA difference (ND5)	Histological grading	1.10	2	0.58
mtDNA difference (ND1) (decreasing)	Histological grading	1.15	2	0.56
mtDNA difference (ND5) (decreasing)	Histological grading	0.32	2	0.85
mtDNA difference (ND1)	Nuclear grading	0.05	1	0.83
mtDNA difference (ND5)	Nuclear grading	0.76	1	0.38
mtDNA difference (ND1) (decreasing)	Nuclear grading	0.97	1	0.32
mtDNA difference (ND5) (decreasing)	Nuclear grading	0.18	1	0.67
mtDNA difference (ND1)	Treatment response	0.01	1	0.90
mtDNA difference (ND5)	Treatment response	0.06	1	0.80
mtDNA difference (ND1) (decreasing)	Treatment response	0.59	1	0.44
mtDNA difference (ND5) (decreasing)	Treatment response	0.10	1	0.75

* *p* is a statistical significance of association, (*p* < 0.05).

**Table 7:** Results of the Kruskal–Wallis test for mtDNA differences for treatment response.

Variable	Grouping Variable	Chi-Squared Statistic	Degrees of Freedom (df)	*p*-Value *
mtDNA difference (ND1)	Tumor T stage	7.98	6	0.34
mtDNA difference (ND5)	Tumor T stage	7.57	6	0.27
mtDNA difference (ND1) (decreasing)	Tumor T stage	4.55	5	0.47
mtDNA difference (ND5) (decreasing)	Tumor T stage	2.67	5	0.75
mtDNA difference (ND1)	Tumor N stage	0.84	6	0.99
mtDNA difference (ND5)	Tumor N stage	1.49	6	0.96
mtDNA difference (ND1) (decreasing)	Tumor N stage	3.80	6	0.70
mtDNA difference (ND5) (decreasing)	Tumor N stage	1.61	6	0.70
0mtDNA difference (ND1)	Stage	2.68	4	0.61
mtDNA difference (ND5)	Stage	2.26	4	0.69
mtDNA difference (ND1) (decreasing)	Stage	4.01	4	0.40
mtDNA difference (ND5) (decreasing)	Stage	2.78	4	0.59
mtDNA difference (ND1)	Histological grading	1.49	3	0.68
mtDNA difference (ND5)	Histological grading	1.69	3	0.64
mtDNA difference (ND1) (decreasing)	Histological grading	1.94	3	0.58
mtDNA difference (ND5) (decreasing)	Histological grading	0.33	3	0.95
mtDNA difference (ND1)	Nuclear grading	0.05	2	0.97
mtDNA difference (ND5)	Nuclear grading	1.11	2	0.57
mtDNA difference (ND1) (decreasing)	Nuclear grading	2.78	2	0.50
mtDNA difference (ND5) (decreasing)	Nuclear grading	0.20	2	0.90
mtDNA difference (ND1)	Tumor size	21.63	20	0.36
mtDNA difference (ND5)	Tumor size	19.77	20	0.47
mtDNA difference (ND1) (decreasing)	Tumor size	3.80	6	0.70
mtDNA difference (ND5) (decreasing)	Tumor size	1.61	6	0.95

* *p* is a statistical significance of association, (*p <* 0.05).

## Data Availability

The data supporting this study’s findings are available from the corresponding author upon reasonable request.

## Supplementary Materials

The following supporting information can be downloaded at: https://www.mdpi.com/article/10.3390/diseases13060175/s1, Table S1: DNA concentration and purity metrics; Table S2: mtDNA content of ND1; Table S3: mtDNA content of ND5.

## Abbreviations

The following abbreviations are used in this manuscript:

CNV	Copy number variations
IDC	Invasive ductal carcinoma
mtDNA	Mitochondrial DNA
ROS	Reactive oxygen species
TNBC	Triple negative breast cancer
qPCR	Quantitative PCR
