# Supplementary material for: Association of Mitochondrial DNA Copy Number Variations with Triple-Negative Breast Cancer: A Potential Biomarker Study
Source: Diseases. 2025 Jun 1;13(6):175. doi: 10.3390/diseases13060175 (PMC12192263; doi:10.3390/diseases13060175)
Supplement: Supplementary file 1 [file diseases-13-00175-s001.zip › diseases-3642517-supplementary.pdf]

Supplementary Tables

The following supplementary tables provide detailed metrics of DNA isolation quality, as well as mtDNA content measurements for ND1 and ND5 in both corresponding normal and TNBC tissues from 23 samples.

Table S1. DNA concentration and purity metrics.

	Corresponding Normal Tissue		Tumor Tissue	
Sample	Nanodrop concentration (ng/µl)	260/280	Nanodrop concentration (ng/µl)	260/280
1	30.00	1.58	118.45	1.81
2	57.40	1.63	273.15	1.95
3	17.65	1.56	31.20	1.67
4	22.00	1.37	9.40	1.29
5	17.75	1.54	108.95	1.90
6	20.50	1.45	40.65	1.70
7	17.45	1.69	47.05	1.77
8	18.95	1.97	64.35	1.72
9	21.90	1.61	49.10	1.78
10	328.50	2.02	34.85	1.79
11	15.60	1.45	36.60	1.63
12	89.25	2.03	53.35	1.78
13	11.55	2.08	67.75	1.77
14	23.45	1.70	544.15	2.06
15	42.45	1.32	20.15	1.37
16	18.45	1.85	53.3	1.74
17	39.40	1.90	135.85	1.89
18	22.30	1.70	96.90	1.85
19	24.80	1.96	10.25	1.28
20	13.60	1.92	44.30	1.95
21	15.40	1.49	34.70	1.68
22	29.45	1.66	45.25	1.86
23	22.55	2.12	108.30	1.84

Table S2 mtDNA content of ND1

	Corresponding Normal Tissue			Tumor Tissue		
Sample	18S rRNA	ND1	2 x $2^{\Delta Ct}$	18S rRNA	ND1	2 x $2^{\Delta Ct}$
1	23.38 ± 0.26	17.71 ± 0.07	101.61 ± 0.17	26.18 ± 0.06	21.72 ± 0.12	43.98 ± 0.09
2	20.29 ± 0.18	18.17 ± 0.13	8.65 ± 0.15	18.67 ± 1.05	17.46 ± 0.03	4.62 ± 0.54
3	24.60 ± 0.39	21.07 ± 0.15	23.03 ± 0.27	24.51 ± 0.03	21.48 ± 0.17	16.33 ± 0.10
4	26.01 ± 0.08	21.65 ± 0.23	41.15 ± 0.16	27.34 ± 0.36	26.71 ± 0.18	3.10 ± 0.27
5	23.76 ± 0.25	20.30 ± 0.05	21.92 ± 0.15	21.12 ± 0.18	18.66 ± 0.03	11.03 ± 0.10
6	23.12 ± 1.32	16.99 ± 0.20	59.45 ± 0.76	21.40 ± 0.43	16.81 ± 0.10	42.59 ± 0.27
7	22.83 ± 0.01	19.26 ± 0.08	23.79 ± 0.05	20.21 ± 0.22	18.80 ± 0.10	5.34 ± 0.16
8	20.03 ± 0.14	17.32 ± 0.30	13.02 ± 0.22	18.69 ± 0.10	16.75 ± 0.12	7.67 ± 0.11
9	22.05 ± 0.04	18.54 ± 0.06	22.91 ± 0.05	22.71 ± 0.09	19.20 ± 0.15	22.76 ± 0.12
10	21.04 ± 0.03	16.99 ± 0.38	33.18 ± 0.20	18.53 ± 0.39	16.67 ± 0.03	7.27 ± 0.21

11	21.83 ± 0.01	18.16 ± 0.09	25.49 ± 0.05	19.71 ± 0.16	17.30 ± 0.08	10.60 ± 0.12
12	20.66 ± 2.89	16.85 ± 0.01	28.09 ± 1.45	19.97 ± 0.14	18.52 ± 0.26	5.47 ± 0.20
13	20.79 ± 0.36	17.32 ± 0.20	22.24 ± 0.28	19.80 ± 0.11	18.16 ± 0.06	6.23 ± 0.08
14	21.42 ± 0.10	17.15 ± 0.04	38.65 ± 0.07	20.21 ± 0.07	16.64 ± 0.13	23.69 ± 0.10
15	23.97 ± 0.07	20.29 ± 0.11	25.6 ± 0.09	23.95 ± 0.33	20.35 ± 0.20	24.21 ± 0.26
16	22.93 ± 0.05	18.98 ± 0.18	30.83 ± 0.11	19.29 ± 0.04	17.17 ± 0.05	8.70 ± 0.04
17	22.17 ± 0.03	17.75 ± 0.05	42.97 ± 0.04	19.43 ± 0.55	16.33 ± 0.08	17.24 ± 0.31
18	24.94 ± 0.43	18.62 ± 0.17	159.71 ± 0.30	20.22 ± 0.17	17.31 ± 0.10	15.06 ± 0.13
19	20.41 ± 0.04	16.73 ± 0.20	25.70 ± 0.12	30.11 ± 0.03	25.38 ± 0.05	53.14 ± 0.04
20	20.09 ± 0.04	16.96 ± 0.12	17.49 ± 0.08	21.43 ± 0.12	17.27 ± 0.10	35.84 ± 0.11
21	22.20 ± 0.10	19.12 ± 0.05	16.91 ± 0.07	21.58 ± 0.27	18.46 ± 0.07	17.34 ± 0.17
22	20.72 ± 0.01	18.21 ± 0.12	11.34 ± 0.06	20.37 ± 0.01	17.69 ± 0.13	12.85 ± 0.07
23	20.68 ± 0.13	17.05 ± 0.02	24.88 ± 0.07	20.40 ± 0.25	16.34 ± 0.05	33.32 ± 0.15

Table S3 mtDNA content of ND5

	Corresponding Normal Tissue			Tumor Tissue		
Sample	18S rRNA	ND5	2 x $2^{\Delta Ct}$	18S rRNA	ND5	2 x $2^{\Delta Ct}$
1	23.38 ± 0.26	21.15 ± 0.93	9.37 ± 0.60	26.18 ± 0.06	26.07 ± 0.19	2.15 ±0.13
2	20.29 ± 0.18	19.85 ± 1.15	2.70 ± 0.66	18.67 ± 1.05	18.59 ± 0.09	2.11 ± 0.57
3	24.60 ± 0.39	22.67 ± 0.02	7.58 ± 0.20	24.51 ± 0.03	25.56 ± 0.01	0.97 ± 0.02
4	26.01 ± 0.08	24.13 ± 0.27	7.39 ± 0.18	27.34 ± 0.36	28.62 ± 0.10	0.82 ± 0.23
5	23.76 ± 0.25	20.30 ± 0.05	21.92 ± 0.15	21.12 ± 0.18	18.66 ± 0.03	11.03 ± 0.10
6	23.12 ± 1.32	19.77 ± 0.24	20.48 ± 0.78	21.40 ± 0.43	20.00 ± 2.85	5.25 ± 1.64
7	22.83 ± 0.01	20.59 ± 0.16	9.44 ± 0.08	20.21 ± 0.22	20.13 ± 0.05	2.12 ± 0.14
8	20.03 ± 0.14	18.00 ± 0.04	8.17 ± 0.09	18.69 ± 0.10	17.30 ± 0.16	5.24 ± 0.13
9	22.05 ± 0.04	20.37 ± 0.06	6.42 ± 0.05	22.71 ± 0.09	21.84 ± 0.74	3.67 ± 0.41
10	21.04 ± 0.03	18.53 ± 0.70	11.37 ± 0.37	18.53 ± 0.39	18.52 ±0.48	2.02 ± 0.43
11	23.20 ± 0.19	20.65 ± 0.16	11.76 ± 0.18	20.49 ± 0.03	19.37 ± 0.08	4.35 ± 0.06
12	19.27 ± 0.01	17.10 ± 0.04	8.98 ± 0.02	20.87 ± 0.07	21.20 ± 0.09	1.59 ± 0.08
13	22.02 ± 0.47	20.27 ± 0.16	6.71 ± 0.32	20.92 ± 0.26	19.87 ± 0.09	4.12 ± 0.17
14	21.42 ± 0.10	18.57 ± 0.10	14.39 ± 0.10	20.21 ± 0.07	18.17 ± 0.12	8.24 ± 0.10
15	23.97 ± 0.07	22.35 ± 0.09	6.13 ± 0.08	23.95 ± 0.33	22.55 ± 0.01	5.25 ± 0.17
16	22.93 ± 0.05	20.19 ± 0.14	13.34 ± 0.10	19.29 ± 0.04	19.90 ± 0.17	1.31 ± 0.10
17	22.17 ± 0.03	20.15 ± 0.45	8.10 ± 0.24	19.43 ± 0.55	18.25 ± 0.17	4.55 ± 0.36
18	24.94 ± 0.43	20.29 ± 0.04	50.35 ± 0.23	20.22 ± 0.17	18.88 ± 0.21	5.08 ± 0.19
19	20.41 ± 0.04	18.56 ± 0.13	7.23 ± 0.08	30.11 ± 0.03	28.02 ± 0.82	8.55 ± 0.43
20	20.88 ± 0.08	20.17 ± 0.09	3.26 ± 0.08	22.19 ± 0.21	19.52 ± 0.06	12.75 ± 0.14
21	23.23 ± 0.16	22.80 ± 0.003	2.70 ± 0.08	22.89 ± 0.07	22.28 ± 0.15	3.04 ± 0.11
22	21.68 ± 0.19	20.02 ± 0.17	6.33 ± 0.18	22.35 ± 0.27	19.73 ± 0.19	12.25 ± 0.23
23	20.68 ± 0.13	18.44 ± 0.26	9.49 ± 0.20	20.40 ± 0.25	16.75 ± 0.11	25.16 ± 0.18
